# Exercise and Bariatric Surgery: An Effective Therapeutic Strategy

**DOI:** 10.1249/JES.0000000000000168

**Published:** 2018-09-14

**Authors:** Paul M. Coen, Elvis A. Carnero, Bret H. Goodpaster

**Affiliations:** 1 Translational Research Institute for Metabolism and Diabetes, Florida Hospital; and; 2 Sanford Burnham Prebys Medical Discovery Institute at Lake Nona, Orlando, FL

**Keywords:** bariatric surgery, exercise, weight loss maintenance, type 2 diabetes, energy expenditure

## Abstract

The long-term efficacy of bariatric surgery is not entirely clear, and weight regain and diabetes relapse are problems for some patients. Exercise is a feasible and clinically effective adjunct therapy for bariatric surgery patients. We hypothesize that exercise is also a critical factor for long-term weight loss maintenance and lasting remission of type 2 diabetes.

## Abstract 2

Exercise is a clinically effective adjunct therapy with the potential to promote long-term weight loss maintenance for bariatric surgery patients.

**Key Points**

- Bariatric surgery can be an effective therapeutic option for obesity.
- The long-term efficacy of bariatric surgery is not entirely clear; weight regain and diabetes relapse are problems for some patients.
- Recent evidence indicates that exercise is a feasible and clinically effective adjunct therapy for bariatric surgery patients.
- Exercise may also be a critical factor for long-term weight loss maintenance and lasting remission of type 2 diabetes.

## INTRODUCTION

Severe obesity is defined as a body mass index (BMI) of ≥40 kg·m^−2^ and is a serious and prevalent health issue in many western countries, including the United States (1). Obesity is now classified as a disease by the American Medical Association (2) and can cause many common adverse health outcomes including type 2 diabetes (T2D), cancer, arthritis, hypertension, and cardiovascular diseases.

Bariatric surgery is a generally safe and effective treatment option for obesity and encompasses a number of different procedures (3). The most commonly performed bariatric surgery procedures in the United States, sleeve gastrectomy and Roux-en-Y gastric bypass (RYGB), result in dramatic weight loss, improvements in peripheral tissue insulin sensitivity, and diabetes remission in a large percentage of patients. A structured exercise program is a feasible and effective adjunct therapy for bariatric surgery patients that elicits additional cardiometabolic benefits compared with those experienced with bariatric surgery–induced weight loss alone (4). Structured exercise increases total daily energy expenditure (TDEE) and improves skeletal muscle mitochondrial energetics, fat oxidation, and insulin sensitivity. It is not clear, however, whether exercise or physical activity (PA) can overcome the “metabolic adaptation” or decreased energy expenditure that occurs with surgery-induced weight loss and have an impact on overall daily energy balance. In recent years, the advent of technology that permits quantitative and comprehensive assessment of nonexercise PA (NEPA) and sedentary behavior underscores the importance of these behaviors in energy balance, weight regulation, and the development or worsening of obesity. These behaviors also likely contribute to outcomes after bariatric surgery.

In this review, we discuss our thesis that exercise or increases in PA can be effective as an adjunct therapy for bariatric surgery patients (Fig.). This is a thesis that still needs to be rigorously tested, particularly in the context of long-term outcomes. We start by identifying some of the shortcomings of the surgical options for obesity treatment, including weight regain and diabetes relapse, and suggest that exercise improves energy balance, a critical factor for long-term surgery-induced weight loss maintenance, and contributes to lasting remission of T2D by improving and maintaining peripheral tissue insulin sensitivity. We draw on evidence from exercise and diet-induced weight loss studies to support our thesis. Finally, we outline the areas that future studies should focus on to generate the next level of evidence that will inform contemporary exercise guidelines for this rapidly growing patient population.

**Figure:**
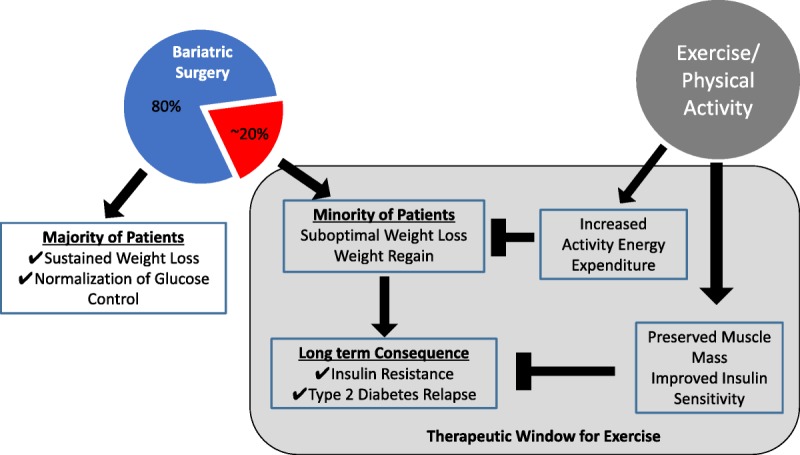
Potential mechanisms by which exercise can impart additional benefit in metabolic health for bariatric surgery patients who experience suboptimal weight loss and weight regain.

## BARIATRIC SURGERY IS NOT A METABOLIC PANACEA FOR ALL PATIENTS

### The Long-term Efficacy of Bariatric Surgery Is Variable

Bariatric surgery is usually an effective therapeutic option for weight loss for severe obesity, as well as for diabetes remission in patients with lower BMI (5). Recent evidence shows that bariatric surgery is more effective than medical therapy alone even out to 5 yr of follow-up (5). Indeed, several professional organizations recently have recommended broadening the indications for bariatric surgery to include individuals with poorly controlled T2D and BMI as low as 30 kg·m^−2^ and down to 27.5 kg·m^−2^ for Asian patients. However, this consensus is based largely on evidence from randomized clinical trials with only 1 to 3 yr of follow-up. The evidence is limited by studies with small sample sizes, insufficient long-term (>1 yr) follow-up in cohorts with adequate retention rates (>80%), and single site studies in the absence of nonsurgical comparison groups (6). Less is known about long-term (>5 yr) weight loss maintenance after bariatric surgery (5,7). In addition, weight regain may be a factor associated with study participant drop out and loss to follow-up, resulting in a skewed and overly optimistic conclusion regarding the efficacy of bariatric surgery–induced weight loss when follow-up is incomplete — less than 80% (6).

Despite of the lack of quality long-term studies, the current evidence suggests that the benefits of bariatric surgery are not universal. Weight regain and diabetes relapse can occur in a significant proportion of patients (8). It has been estimated that 10%–30% of bariatric surgery patients experience suboptimal weight loss (defined as either ≤50% or ≤40% excess body weight loss after gastric bypass (GB) surgery (9)). Indeed, suboptimal weight loss and weight regain are key factors linked to diabetes relapse (8), which can occur in 20%–30% of patients who achieved remission 5 yr after bariatric surgery (8). Recent evidence from the Surgical Treatment and Medications Potentially Eradicate Diabetes Efficiently (STAMPEDE) trial, however, suggests that improvements in glucose control (HbA1C <6% without using medication) are consistent 5 yr after surgery (89% of patients were at least HbA1C <7%) (5). In addition, a proportion of bariatric surgery patients also experience attenuation in the recovery from other comorbidities (after 6 yr, 13%, 11%, and 4% of patients for T2DM, hypertension, and LDL-cholesterol, respectively) (10,11). However, a lack of high-quality long-term data makes it difficult to estimate effects on many comorbidities (6). The question of whether bariatric surgery should be regarded as an effective “cure” for diabetes has been raised (12); in a 12-yr follow-up study, a 51% remission rate of T2DM was observed (13); however, questions of why weight regain and diabetes relapse occur in some patients and not others remain.

### Why Do Some Bariatric Surgery Patients Experience Suboptimal Weight Loss, Weight Regain, and T2D Relapse?

The problem of suboptimal weight loss, weight regain, and T2D relapse is increasingly being recognized, and further investigation is needed to understand the physiological and behavioral origins of interpatient variation in surgery-induced weight loss. Current evidence indicates that greater BMI, age, diagnosis and duration of T2D, cognitive function, personality, and mental health are strong predictors of suboptimal weight loss and diabetes relapse. For example, Brethauer *et al*. (8) demonstrated that diabetes duration and weight regain are major contributors to diabetes relapse after bariatric surgery.

From the perspective of energy balance, a reduced TDEE per kg of fat-free mass (FFM) could compensate for bariatric surgery–induced caloric restriction, which could underlie the variation in weight loss and predispose weight regain. This metabolic adaptation, or hypometabolism, that occurs with weight loss includes changes in resting metabolic rate (RMR), diet-induced thermogenesis (DIT), and PA-associated EE (the main components of TDEE), has not been studied after bariatric surgery.

RMR is determined by total body mass, primarily FFM, and so the loss of adipose and particularly lean tissue mass after bariatric surgery means that less energy is required to sustain resting metabolism. Furthermore, weight loss–induced adaptive thermogenesis results in lower energy expenditure per kilogram of lean tissue than what would be expected. In other words, the body becomes more efficient at using energy. Two mechanisms may explain this adaptation in humans; first, the loss of the different components of FFM mass (internal organs and skeletal muscle mass) does not happen with the same proportion or at the same rate. In this regard, the gastrointestinal system (GI) has a high resting energy demand, up to 10% of body oxygen consumption, and so GI resection (gastric sleeve for example) is likely a mechanism for a reduction in RMR per lean tissue mass.

The alterations in RMR after bariatric surgery have been described in three phases: the first is an elevated RMR that occurs immediately after surgery (14); a second phase of adaptive thermogenesis is evident between the third and sixth month postsurgery (15); and third, the adaptive thermogenesis typically disappears after the first year postsurgery (15). The degree of adaptive thermogenesis has been suggested to be related to the degree of energy balance, so patients who are in energy balance have suppressed adaptive thermogenesis (15). In addition, evidence from animal studies suggested that these dynamic alterations in RMR are likely influenced by the specific surgical procedure (16). However, human studies do not demonstrate significant differences after 6 and 12 months between gastric and vertical banding (−24% and −25%) and RYGB (−19% and −19%) (17,18), although the changes after 12 months were not entirely dependent on body composition changes either in adults (17) or adolescents (18). Additional studies with a precise and accurate assessments of energy intake and energy expenditure would help to clarify the roles of energy balance and adaptive thermogenesis independently of magnitude of weight loss (15). In this sense, PA may contribute to negative energy balance, as long as energy expenditure associated with PA is higher than the PA-associated reduction in RMR (compensation). In summary, future studies should investigate the role of adaptive thermogenesis on alterations in RMR to further understand its role in weight loss response and duration to define clinical relevance.

## EXERCISE AS AN ADJUNCT THERAPY

### Bariatric Surgery Patients Engage in Very Low Daily Physical Activities

Few studies have objectively measured PA and sedentary behaviors in bariatric surgery patients using activity monitors. Recent work from our group and others (19,20) indicates that after bariatric surgery, patients following an exercise intervention or health education controls have low daily PA levels relative to recommendations (<7500 steps per d, <150 min·wk^−1^ in bouts of 10 min or 60–90 min·d^−1^ of moderate PA (20)) and compare with nonobese controls (21). One of the most thorough examinations of PA behaviors after bariatric surgery to date comes from the Longitudinal Assessment of Bariatric Surgery-2 study (LABS-2), wherein 310 participants wore activity monitors for ≥10 h·d^−1^ for ≥3 d pre- and 1-yr postsurgery (22). Overall, these participants increased spontaneous PA by an average of 1457 steps per d, although there was a wide variation in change (from 7648 fewer steps per d to an increase of 17,205 steps per d). Furthermore, only 11% reached ≥150 min·wk^−1^ of PA after surgery. This is far below the 150 min·wk^−1^ of exercise that the American Diabetes Association and American College of Sports Medicine recommend for optimal health. In addition, and depending on the PA parameter, between 23.6% and 29% of participants were less active 1-yr postsurgery. We recently reported that even low levels of PA (7885 to 4343 steps per d for the highest to the lowest quartile) are related to beneficial changes in body composition and insulin sensitivity after bariatric surgery (19,23). These data suggest that a majority of patients are not increasing PA and some even decreasing PA (change in steps per d after 6 months −1419, 406, 1618, and 3446 from the lowest to the highest quartile) (19). Altogether, these previous studies that objectively measured PA suggest that a high proportion of patients are inactive (94% do not meet the recommendations) and more than two thirds are not involved in any bouts of moderate-to-vigorous physical activity (MVPA; 41.3%–49.4%) (24); although these numbers may differ across countries (25), this implies that strategies to increase structured exercise or daily PA levels, or alternatively strategies to lower sedentary behaviors, may have therapeutic value in bariatric surgery patients.

### Is Presurgery Exercise a Viable Therapeutic Approach?

A common belief exists, even in contemporary articles, that lifestyle intervention approaches including exercise are ineffective for treatment of persons with severe obesity (26). Part of the reason for this perception is that there have been very few well-controlled exercise trials in severely obese bariatric surgery patients (27). Clinical practice guidelines for the perioperative support of the bariatric surgery patient include recommendations to increase PA to optimize surgical outcomes. A number of studies (summarized in Table 1 (28–30)) have examined PA interventions before surgery and found that they enhance quality of life, lower feelings of embarrassment during PA, and improve physical fitness. For example, Baillot *et al*. (31) showed that a 12-wk presurgical supervised exercise training program improved 6-min walk time, muscle strength, and physical fitness. To our knowledge, there are only two preoperative PA interventions where participants were followed over 6 months (29) and 12 months after surgery (32); their results confirmed that patients who underwent PA intervention increased their MVPA and steps per d after intervention (Table 1); in addition, their steps per day were significantly higher after surgery than patients receiving standard care (29). However, further evidence is required to clearly define the clinical benefit of PA before surgery on clinical outcomes.

**TABLE 1:**
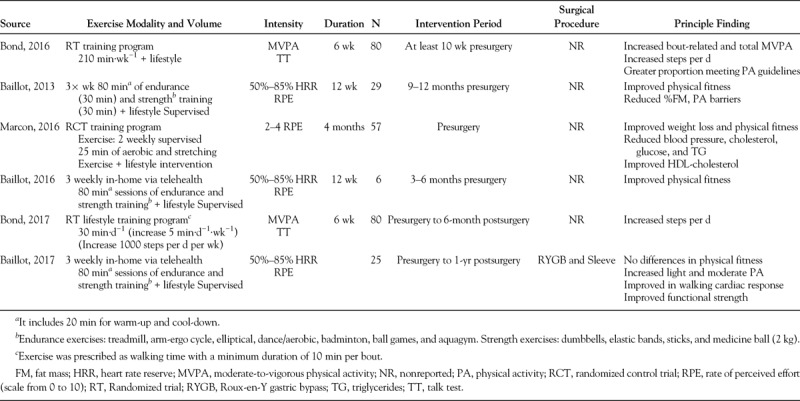
Exercise intervention studies before bariatric surgery

### Is a Postsurgery Exercise Program Feasible and Effective for Severely Obese Bariatric Surgery Patients?

There are currently no evidence-based PA guidelines specifically for bariatric surgery patients. However, recommendations for PA have been made by a number of organizations, including the American Society for Metabolic and Bariatric Surgery (ASMBS), the Obesity Society, and the American Heart Association (20). Common guidelines issued by the ASMBS, the Obesity Society, and the American Association of Clinical Endocrinologists recommend that postoperative patients should adhere to a healthful lifestyle including exercising for at least 30 min·d^−1^ (20). There is currently a dearth of evidence on which to base bariatric surgery–specific PA guidelines.

Our group recently reported that 91% of RYGB patients randomized to a 6-month exercise program successfully completed the intervention. We also found that two thirds of the patients adhered to the *a priori* defined exercise prescription of >120 min·wk^−1^ aerobic training (mainly walking) and exercised an average of 185 min·wk^−1^, which is above the current recommendations outlined in the PA guidelines. In addition, the participants in the exercise group improved cardiorespiratory fitness (absolute (L per min) and relative (L per min per kg) V˙O_2_ peak), such that more minutes of exercise were associated with higher increases in V˙O_2max_ (33). This is a clinically important finding, because cardiorespiratory fitness is associated with a reduced risk of all-cause mortality and other cardiovascular comorbidities. Therefore, not only is an aerobic exercise training program feasible in this patient population, it is also efficacious at improving cardiorespiratory fitness, a result that directly counters the perception that severely obese individuals cannot respond to or will not adhere to a lifestyle or exercise intervention. This study was the first proof of efficacy trial to show that an exercise intervention *per se* can improve health outcomes in bariatric surgery patients. Further research is needed to determine how effective such an exercise program might be in real-world terms, for example, in terms of integration into a clinical bariatric surgery practice.

### Does Exercise Contribute to Greater Weight Loss After Bariatric Surgery?

Exercise alone typically results in weight loss of less than 3% of initial body weight. This often leads to a perceived lack of health benefit of exercise by obese patients in the absence of appreciable weight loss, despite its physiological and psychological health benefits independent of weight loss. However, exercise administered in combination with diet-induced caloric restriction results in significantly greater reductions in body weight (−8.4% vs −11.4% for men and −5.5% vs −7.5% for women after 4-month workout period (34)), even in patients with severe obesity (10.9 kg vs 8.2 kg over a 6-month intervention (35)). Currently, there is scant equivalent evidence for this effect of exercise with bariatric surgery patients. We observed that a short-term (6 months) exercise program did not promote additional weight loss after RYGB (22.0 kg vs 22.8 kg conventional treatment versus 3-month exercise program respectively (4) (see Table 2 for exercise prescription (7,36–41))). These findings are similar to those of Shah *et al*. (27) who showed that a high-volume exercise prescription (>2000 kcal·wk^−1^ at 60%–70% V˙O_2max_) after bariatric surgery had no impact on body weight and waist circumference (4.2 kg and 3.7 cm) when compared with a control group (4.7 kg and 3.6 cm). The lack of an exercise effect on weight in these intervention studies is likely due to the strong influence of surgery as well as the large variability in weight loss. Reports on the mean weight loss induced by surgery with or without exercise do not account for the possibility that a higher dose or intensity of exercise may elicit additional weight loss or alter body composition or regional adiposity in a favorable way after surgery. For example, we reported that patients who performed higher (286 ± 40 min·wk^−1^) amounts of exercise post–bariatric surgery had greater weight and body fat loss compared with those who performed less exercise (42). Aerobic exercise is particularly effective at reducing visceral adipose tissue (VAT) (43), a fat depot that is strongly linked to hepatic insulin resistance (IR) and T2D. It is also plausible that exercise could be particularly effective at eliciting additional weight loss in patients who are experiencing suboptimal weight loss after surgery. Further studies are needed to examine whether higher dose, frequency, or intensity of exercise can further loss of weight or fat mass in bariatric surgery patients.

**TABLE 2:**
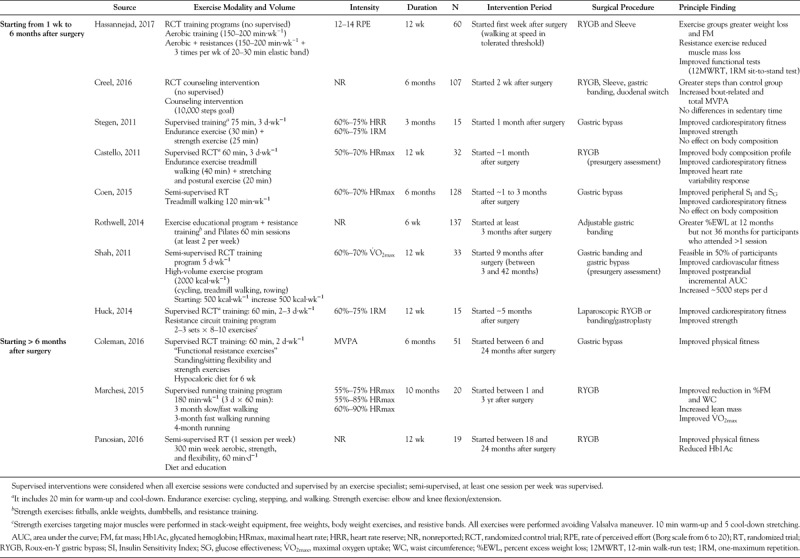
Exercise intervention studies in bariatric surgery patients

### Is Exercise Important for Weight Loss Maintenance?

A significant number of bariatric surgery patients also experience weight regain and reoccurrence of comorbidities (13). For example, a recent report by Adams *et al*. (13) indicated that the mean change from baseline in body weight in a group of 388 surgery patients was −45.0 kg (mean percent change, −35.0) at 2 yr, −36.3 kg (mean percent change, −28.0) at 6 yr, and −35.0 kg (mean percent change, −26.9) at 12 yr, indicating a slow regain of weight by patients. Maintaining weight loss is a well-recognized problem for patients who try diet-induced calorie restriction to lose weight (nonsurgical), with reports suggesting that 12–18 months after weight loss, 33%–50% of initial weight loss is regained (37). Exercise has proven to be an important factor for long-term weight loss maintenance after calorie restriction (44). An examination of data from the National Weight Control Registry indicates that moderate-intensity exercise is critical for maintaining (nonsurgical) weight loss. Intervention trials corroborate this. For example, an exercise intervention study by Jakicic *et al*. (44) indicates that the addition of 275 min·wk^−1^ of PA in combination with a reduction in energy intake was necessary for maintenance of a 10% weight loss in overweight women. The importance of higher doses of exercise to maintain weight loss also has been reported (34). Unfortunately, comparable evidence has not been generated from bariatric surgery patients, and whether exercise may assist weight loss maintenance after surgery is not yet clear. Further long-term exercise intervention trials are now needed to determine whether exercise is important to prevent weight regain in bariatric surgery patients.

### Exercise May Counteract the Physiological Adaptations That Occur With Bariatric Surgery–Induced Weight Loss

FFM accounts for a significant portion of RMR, and FFM loss may predispose weight regain in the long term (45). Indeed, it has been suggested that loss of FFM (skeletal muscle, bone, and organs) accounted for 31.3% of weight loss with RYGB surgery (46). The clinical significance of FFM loss on successful weight loss and propensity for weight regain has not been investigated adequately, but excessive FFM loss is likely undesirable. In addition, for the older bariatric surgery patient, the loss of skeletal muscle and bone density may have a negative impact on physical function, progression of sarcopenia, and quality of life (47). Bariatric surgery–induced weight loss results in significant reductions of bone mineral content (BMC) (48), which is likely relevant for older patients who are at risk for osteoporosis.

Exercise, particularly resistance exercise, can help to attenuate muscle mass loss in RYGB patients (19). In the context of diet-induced calorie restriction, a number of randomized studies have shown that during weight loss, supervised exercise prevents the loss of FFM (49). However, the long-term significance of exercise-induced preservation of muscle mass during calorie restriction on incident mobility disability and physical function has yet to be studied. A 10-month running intervention conducted 1–3 yr after surgery demonstrates that RYGB patients can gain FFM (7). There is a lack of evidence regarding the specific type of exercise (resistance, aerobic or concurrent training), intensity, or dose (number of exercise bouts per day or week) that might be most effective. To our knowledge, there are only two studies that have examined the effect of resistance training after bariatric surgery, and neither study found significant differences in FFM between resistance training (50) or resistance and endurance training (51). Finally, the effect of exercise training in preserving BMC has not been studied after bariatric surgery with older patients (48). The potential for exercise training to effectively prevent or attenuate BMC reductions after bariatric surgery remains to be elucidated.

The influence of exercise training or PA modification on TDEE components has not been examined extensively (52). An elevation in TDEE would stem logically from increasing either PA or exercise training. DIT has received little attention (53), and although it has been associated with longer term weight loss (53), little is known about the specific effects of exercise or PA on DIT. The effects of exercise or PA on activity-related energy expenditure (PAEE) are important but have received little attention. Even if PA increases with a structured exercise program, PAEE can still decrease with weight loss. This may suggest the change in PA-induced EE may not be enough to compensate for the loss of lean body mass and adaptive thermogenesis-associated EE reduction, even after implementing an exercise training program after surgery. This is supported by an inverse relation between RMR and PA in morbidly obese patients who were enrolled in exercise training programs (15,54) and by a recent analysis from our group (15).

The influence of exercise training or lifestyle PA modifications on RMR has not been examined to date in bariatric surgery patients (52). Several studies have performed simultaneous measurements of PA and RMR (19,52), although none of them had analyzed the influence of PA or exercise on adaptive thermogenesis. Inferences with exercise interventions are complicated. Exercise can further reduce weight, lean body mass, and RMR, thereby potentially exacerbating adaptive thermogenesis. Previous studies in severely obese patients who were enrolled in exercise training programs have shown an inverse relation between RMR and volume of PA (15,54), which could suggest a constrained total energy expenditure to PA paradigm. Conversely, resistance training has been shown to preserve RMR independently of body composition changes (55) and this could be beneficial after bariatric surgery. This, however, is speculative and requires further investigation.

### Important Distinction and Potential Interplay Between PA and Exercise

The most variable component of TDEE is PA-associated energy expenditure, which could be simply split into exercise-related energy expenditure (associated with exercise training) or nonstructured PA energy expenditure (associated with intentional and nonintentional PA). Implementing exercise training programs may increase EE per minute during the exercise period, but there is controversy in the literature about the effects of exercise training on total daily PA behaviors, and it has been suggested that exercise interventions can cause compensatory reductions in nonexercise activity, thus reducing total daily PA and EE (19). This could in effect create a resistance to weight loss. In a recent study from our group (19), we suggest that RYGB patients enrolled into a structured exercise program may reduce their NEPA after 6 months of aerobic exercise training with an average volume of 185 min·wk^−1^ (mainly walking) at 50%–70% of the individual maximum heart rate.

### Improvement in Peripheral IR May Be Key for Lasting T2D Remission

IR refers to the blunted response of a tissue to circulating insulin, and peripheral tissue IR is a key early factor in the development of T2D. In the days after bariatric surgery, the acute caloric restriction that occurs improves hepatic insulin sensitivity and glucose control (37). Anatomical changes due to the RYGB procedure (shorter Roux limb) also contribute to a greater incretin response, which is also thought to improve glucose control immediately postsurgery (37). Studies using the glucose clamp with stable isotopic tracers confirm that endogenous glucose production, an indicator of hepatic insulin sensitivity, improves soon after RYGB surgery (56). Studies using the glucose clamp method also reveal that the immediate metabolic benefits of bariatric surgery do not extend to improvements in peripheral tissue insulin sensitivity (56). Indeed, a report showed that 1 month after RYGB surgery and substantial weight loss (~11%), peripheral insulin sensitivity did not improve (56). This is significant, however, as skeletal muscle is the primary peripheral tissue responsible for disposal of ~80% of glucose after a meal. The long-term improvements in peripheral tissue insulin sensitivity after bariatric surgery occur in proportion to weight loss (57), which typically consist of a ~50% reduced whole body fat mass and a ~60% decrease in VAT after 1 yr (58). However, even with significant weight loss 1 yr after RYBG surgery, peripheral insulin sensitivity remains low compared with lean metabolically healthy individuals (37).

It is plausible that the lack of normalization of IR may leave bariatric surgery patients prone to relapse of diabetes. IR is a key feature of prediabetes and strongly predicts the future occurrence of T2D in nonsurgery patients. Diabetes duration and weight regain are contributors to diabetes relapse (8), and the degree of improvement of peripheral IR (and maintenance of improvement) and retention of glucose control likely influence the future risk of diabetes relapse. Indeed, epidemiological studies of nonsurgery patients have demonstrated that oral glucose tolerance test (OGTT)-derived measures of IR can predict future development of T2D (37). In this context, exercise may be beneficial to improve peripheral tissue insulin sensitivity after surgery-induced weight loss and perhaps prevent T2D remission. This hypothesis needs to be tested.

Clinical trials have shown that in individuals at high risk for T2D, a moderate increase in PA reduced the conversion rate of impaired glucose tolerance to T2D by 58% (37). A number of reports recently describe how exercise training after bariatric surgery provides additional improvements in glycemic control (Table 2). Shah *et al*. (27) reported that a 12-wk exercise intervention after RYGB and gastric banding surgery improved glucose tolerance. Results from our randomized controlled trial indicate that moderate aerobic exercise elicits additional improvements in insulin sensitivity and glucose effectiveness, that is, the ability of glucose *per se* to facilitate glucose disposal, along with improved cardiorespiratory fitness during RYGB surgery-induced weight loss (4).

## FUTURE DIRECTIONS

There are a number of pertinent questions that remain germane to the clinical and physiological role of exercise after bariatric surgery. For example, the fine details of what is a feasible and effective PA intervention after surgery, in terms of dose (duration and intensity) and modality (walking, swimming, and cycling), need to be determined. After all, efficacy and effectiveness of exercise and PA to promote health benefits are both distinct but important issues. Does increased exercise/PA provide additive weight loss or fat mass loss, particularly visceral fat or hepatic fat, after bariatric surgery and is it an important factor for long-term weight loss maintenance after bariatric surgery? For the older bariatric surgery patient, there are a number of specific concerns: does the loss of lean mass, including muscle and bone, after surgery may have detrimental consequences on physical function and mobility in older adults? Does exercise after bariatric surgery improve muscle function, reduce osteoarthritis and knee pain, and improve quality of life in older adults?

## SUMMARY

Obesity, severe obesity, and associated comorbidities are major health care problems in the United States and worldwide. Bariatric surgery is likely the most effective treatment option for many with severe obesity, but the benefits are not universal to all patients. In addition, the long-term (>1 yr) effectiveness of bariatric surgery remains unclear. Exercise clearly elicits a multitude of beneficial health effects. We now have objective evidence that PA in patients before and after their bariatric surgery is very low and indeed does not increase substantially during and after weight loss. Hence, this is a patient population who may benefit greatly from increased exercise or PA (Fig.). Randomized controlled trials are needed to more clearly define the exercise dose/intensity needed to provide additional health benefits after bariatric surgery. This valuable evidence also will inform clinical practice regarding much-needed guidelines for exercise after bariatric surgery and will help elucidate the mechanisms by which these improvements are mediated.
